# Standardizing continuous data classifications in a virtual T-maze using two-layer feedforward networks

**DOI:** 10.1038/s41598-022-17013-5

**Published:** 2022-07-27

**Authors:** Johannes Rodrigues, Philipp Ziebell, Mathias Müller, Johannes Hewig

**Affiliations:** Julius-Maximilians-Universität Würzburg, Würzburg, Germany

**Keywords:** Cognitive neuroscience, Psychology, Human behaviour, Machine learning

## Abstract

There continues to be difficulties when it comes to replication of studies in the field of Psychology. In part, this may be caused by insufficiently standardized analysis methods that may be subject to state dependent variations in performance. In this work, we show how to easily adapt the two-layer feedforward neural network architecture provided by Huang^1^ to a behavioral classification problem as well as a physiological classification problem which would not be solvable in a standardized way using classical regression or “simple rule” approaches. In addition, we provide an example for a new research paradigm along with this standardized analysis method. This paradigm as well as the analysis method can be adjusted to any necessary modification or applied to other paradigms or research questions. Hence, we wanted to show that two-layer feedforward neural networks can be used to increase standardization as well as replicability and illustrate this with examples based on a virtual T-maze paradigm^2–5^ including free virtual movement via joystick and advanced physiological data signal processing.

## Introduction

Since Ioannidis^1–6^ as well as the open science collaboration under Nosek and colleagues^7^ pointed out that psychology is facing a massive problem concerning replication of research studies, the focus on reliable and replicable analysis as well as replicable methodological approaches grows stronger. The open access documentation of methods and analysis are becoming more and more common these days in Psychology, which is of key importance in order to get to reliable and replicable results. However, as the focus lies primarily on the documentation and standardization of the data analysis, one may forget about the problems arising in complex data patterns classification. Examples for those kinds of data would be continuous movement recordings (e.g., mouse trajectory, joystick movement, virtual movement in virtual reality, driving simulation, head movement, animal movement trajectories) but also continuous physiological recordings (e.g., human and animal EEG, EMG, ECG, EDA, MRI) and complex combinations of distinct variables (e.g., digital picture recognition). In rather simple cases, the needed replicable classification can be achieved using simple logical and algebraic expressions (e.g., in a force choice task where one knows the alternatives, in movement recordings where one distinct feature such as “going up” is identifying a behavioral class “going to floor 2” for all participants, because they all start on floor 1 and there is no floor 3). Since this simple structure is not always given, complex behavioral patterns are often classified by human experts. These experts may introduce subjective and non-replicable biases into the data, as they are not available to everyone and possibly also sometimes having a different detection threshold concerning underlying patterns in the data due to decreased performance because of stress or other obstacles and states^8–10^. In order to overcome this problem, some researchers started to use neural network pattern classifier to get to a reliable and replicable result (e.g., classification of sleep stages^11–16^, selection of artifact ICs in EEG research^17–19^).

However, neural networks are not generally “better” than humans in decision making. The idea of neural networks especially pattern classifier being an entire new and better level of decision making and classification is sometimes propagated, but very often this is not the case (c.f. neural nets that outperform humans^20,21^). The major reason for humans outperforming neural networks, at least in case of the classical pattern classifiers, is that the classifier has to learn from data and must be trained by data^22^. Hence one already has to know what is right or wrong, ergo one has to previously classify the items of interest and train this classification to the pattern classifier. This may lead to the result that the neural network is not performing better than the human decision maker or expert normally. However, it is more consistent, as the underlying decision rules of the classification will hopefully be discovered by the pattern classifier and used accordingly for the new data. Hence, they are more consistent and therefore they may introduce less variability in the data. Also, they can be made available for everyone, which is hard to be achieved for or by human experts. Still, in the field of psychology many decisions are made by the experts and the replacement by automatic detection algorithms is not yet implemented in many cases of complex behavioral decisions, because it is rather hard to achieve sufficient performance with simple automated algorithms. Nevertheless as mentioned above, some fields successfully implemented neuronal nets in order automatize decisions that were previously made by hand^11–19^. Also, the contributions of deep learning in solving complex behavioral decisions are advancing^21,23,24^. Combined with big data, these approaches are promising concerning complex applications of any sort. Yet, in the field of psychology in many cases, the simple two-layer feedforward network may suffice to solve the classification problem at hand and may need much less computational and applicational effort^23,25^. If the decisions are still made by hand however, even by experts, this leads to the problem that only the researcher themselves or the expert might reproduce the decisions. Concerning the reliability of the decisions, this leads to disastrous effects, especially if the decision is not that easy for a given task set^10,26,27^ and one has to decide between categories that may not be separated easily. For example, this has been shown in a categorization task involving fish outlines that varied across four different shape dimensions, namely “dorsal fin”, “tail”, “ventral fins”, and “mouth”, which might theoretically seem easy to separate without difficulties, but empirically were not^28^. Here, a neural network might provide a good solution for reproducible and reliable effects, although it is not necessarily more accurate than a human decision maker^20^, but still it might be more consistent. Another great feature of neural networks is the generalization of the decisions even to problems that were not present before^21–23^. This feature can lead to a good classification of data that are not learned but similar in their structure to the training data. Accordingly, neural networks provide a mechanism that will generalize the classification to new stimuli, although one has to admit, that we humans tend also to be able do that. However, this neat feature of learning a decision pattern that can generalize comes at a price. If the data is not similar, the neural network will still classify it according to its internal rules and propagate a data classification, although it might not be correct. So, if the data is not similar to the data that was used to train the neural net, there can be problems with the classification, and we will need a new net in order to classify the data correctly. On the other hand, as there are many similar problems or similar outcome classifications, one might use one net for different classification tasks as long as the output and input is similar. Nevertheless, for different tasks, different neural networks are needed^23,25^. So, if we need many different neuronal networks to classify our data, how do we construct the neural networks in order to solve the classification problems? Here we can get very different answers^29^, but we would like to focus on work by Huang^1^, that showed that in theory, any number of distinct samples can be learned with two-layer feedforward networks and therefore, with a training set representing the problem in an exhaustive way, every classification problem can in theory be solved with two-layer feedforward networks. However, it has been shown that very complex problems are solved better by other types of architectures, especially deep learning approaches including recurrent structures^23^ or multilayer networks, especially for modeling visual pattern recognition^21^Yet, for simpler tasks, the two-layer feedforward networks are a good tradeoff between computational resources and construction complexity. The learning capacity of the two-layer feedforward networks was described by Huang who provided a formula how to construct these networks and this formula was used to create the networks presented in this manuscript. In the following, we will show which criteria were used to create the neural network for classification and which training and performance parameters were extracted. Additionally, we will briefly introduce the solution and the overall fit of the solution that was tested with other data. However, the fit of the neural network is not the only indicator that is worth having a look at, but also the performance on a new data set, because some algorithms tend to find an overly specific solution to the problem (overfitting) and tend not to generalize to the problem as well as other networks^30–32^.

Before we start to explain the problems that we used as examples for the classification of the two-layer feedforward network in the “Methods” section, we take a brief look on what this type of network is. A neural network basically consists of different units or neurons, which are put in different layers and will eventually be linked according to different learning rules and propagation constraints in order to propagate the input information to the output layer^22,23^. The weights, the strengths of the links between neurons in one layer to the next layer, store the information and the learned feature association in the net. Every net has an input layer and an output layer, but between them can be a variable amount of neuron layers. These layers between the input and output layers are called hidden layers. Depending on the number of layers and the amount of neurons in the different layers compared to the following layer, the net reacts systematically different to the input. In general, if few layers are used, a very high amount of neurons are needed per layer as the different features and information of the input need to be classified by these layers and their interconnection using the provided learning rule and the propagation constraints. In our case, we are using a two-layer feedforward network. This means, that we are using two hidden layers between the input and the output layer, additionally to the constraint that the data is only propagated from the input layer to the output layer through the two hidden layers without any loop as one would have with for example recurrent or backpropagation of the data^22,23,33^. A graphical display of the two-layer feedforward networks that are used as examples are given in Figs. 4 and 5 in the “Methods” section below.

Subsequently, we want to shed light on our motivation to provide examples for the use of these networks. The reason why we want to provide the community with those examples is that these solutions solve the problem of replicable results as well as accurate classification of rather complex problems that are solvable by using an exhaustive training set, because the neural networks that are constructed mirror the decisions of the experts in these cases. Although one has to mention, that the two-layer feedforward net is not the best architecture to solve very complex tasks (c.f. image recognition^20,34^), the tasks mentioned below are very well solvable by this type of network. Hence, one will be able to create efficiently different neural networks for other tasks, mirroring expert decisions concerning this task. Therefore, a new level of standardization in complex tasks as well as a new level of scientific discussion may arise based on different neural networks that can be applied to datasets of different working groups. We want to provide other researchers with the tools for the classification of these moderately complex tasks that normally need expert decisions as no simple algorithmic solution was at hand, and with the necessary knowledge on how to build a new automatized classification network for their own specific research questions. Especially in the field of psychology, where a replication crisis has been identified in the last few years^35,36^, the sharing of expert knowledge by creating neural networks trained by experts can help to overcome this crisis, by providing expertise even to undergraduate researchers^36^. As the field of psychology is rather diverse, section specific applications and paradigms, problems and the corresponding knowledge to solve them is part of the field of psychology. Hence, the ability to transfer this knowledge to other researchers using neural networks may help in avoiding replication failure. Yet, many problems analyzed in the field of psychology may not be as complex as to require utilizing more powerful computational approaches that are needed to solve very complex problems^20,22,23,34^. Accordingly, the present study is an application of the procedure suggested by Huang^1^ and just one example of its applicability to behavioral classification and physiological data classification (for details see “Methods” section below). One may use such networks to categorize many moderately complex problems where an expert is normally needed and given, as well as a reasonable input and output for the neural network can be defined. At the end of the manuscript, we further discuss the implication of our approach and the opportunities that are given by implementing neural networks as provided in this manuscript, especially concerning the replication crisis and future development of the field of psychology in developing scientific standards and standardization.

In order to further share our knowledge with the scientific community, we additionally provide the environment of our current examples of complex research problems that we address here as an example. Thus, we offer other working groups the opportunity to use and further develop the behavioral classification solution or the physiological data classification along with the paradigm. The environment is a virtual T-maze developed by Rodrigues^2–5^. It was used for recording psychophysiological signals such as the electroencephalogram, electrodermal activity and heart rate while showing different virtual free-choice behavior patterns via joystick in different situations. These behavioral decisions and performances were previously categorized by hand according to standardized criteria^2–5^ into six categories. However, as small changes in the paradigm led also to changes in the resulting behavior, six new categories were proposed and a previously trained neural network to classify the behavior^5^ was not of good use anymore. A detailed description of the different categories is mentioned below in the “Methods” section and Fig. 2. Importantly, based on these classifications or any other behavioral or physiological categorizations, one is able to provide a dataset that can be used to train a neural network in order to automatize and standardize the classification of behavioral or physiological responses (here as an example in the virtual T-maze). In order to provide other researchers with the necessary tracking devices for the joystick movement in the present example of behavioral responses in the virtual T-maze, a tutorial will be attached in the supplementary materials, describing how to set up the paradigm correctly and how to use the free trial version of the tracking software that was used to record the behavioral responses in the virtual T-maze. With this work we hope to support and encourage other researchers to share their paradigms as well as to develop standardized ways for analyzing even difficult phenomena or data. Furthermore, we try to provide the scientific community with our paradigm and the necessary tool to analyze data from it. It may be used as an example to share and it can be modified to the needs of the researchers and the solution for automatized classification (for example in this paradigm) may lead to replication, along with further development of research paradigms.

## Methods

### Ethical statement

The data collection was carried out in accordance with the recommendations of the “Ethical guidelines, The Association of German Professional Psychologists” (“Berufsethische Richtlinien, Berufsverband Deutscher Psychologinnen und Psychologen”) with written informed consent from all subjects All subjects gave written informed consent before they participated in the experiment. The study was conducted in accordance with the Declaration of Helsinki. The study protocol was approved with the file reference GZEK-2017-18 by the local ethics committee of the department of psychology of the Julius-Maximilians-University of Würzburg (Ethikkommission des Institutes für Psychologie der Humanwissenschaftlichen Fakultät der Julius-Maximilians-Universität Würzburg).

### Classification problem example 1: behavior in virtual T-maze paradigm^2–5^

**Figure 1:**
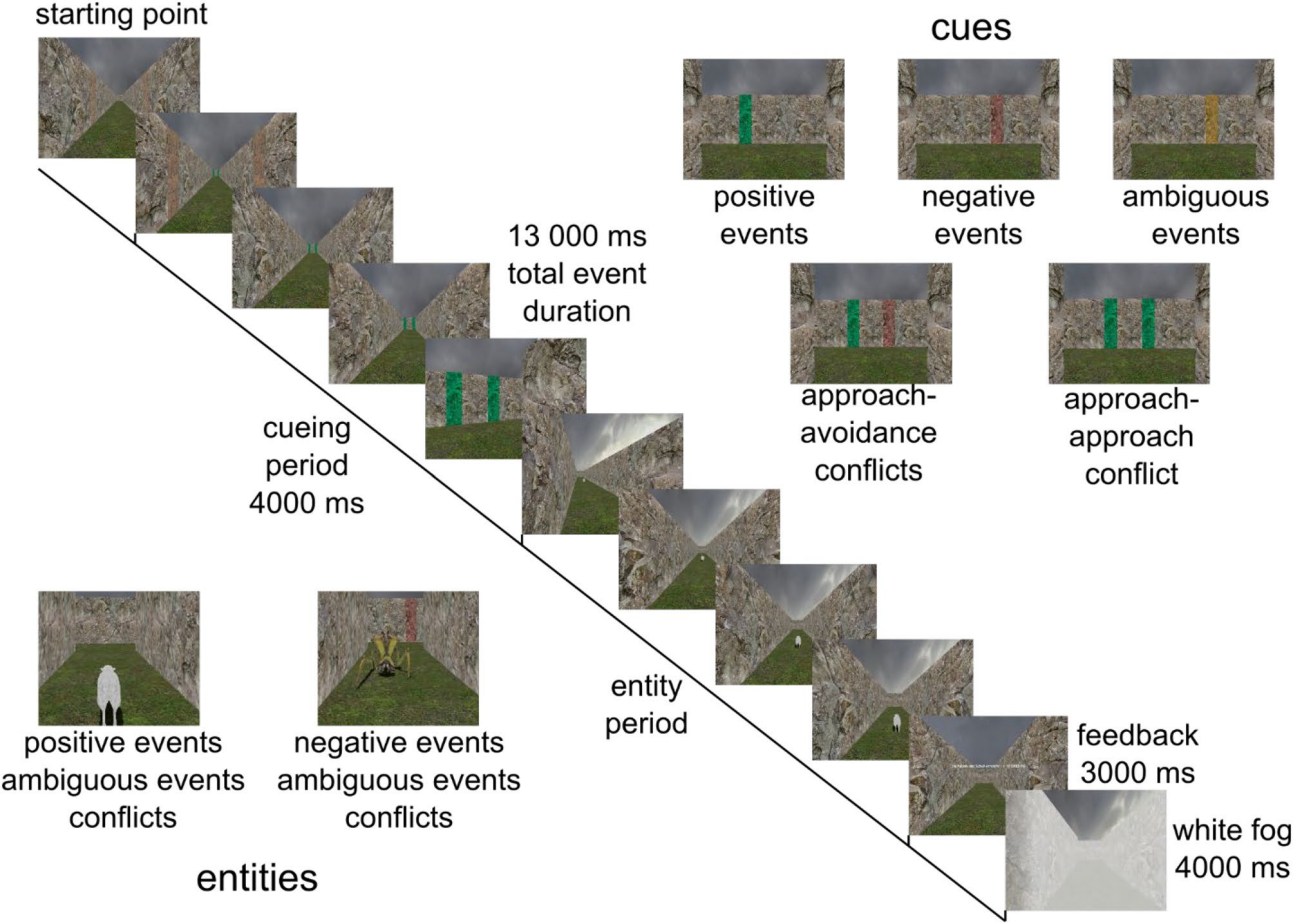
Schematic display of a trial in the VR paradigm. In the lower left corner examples of the entities used in the VR paradigm, in the upper right corner examples of the cues used in the trials are shown.

**Figure 2:**
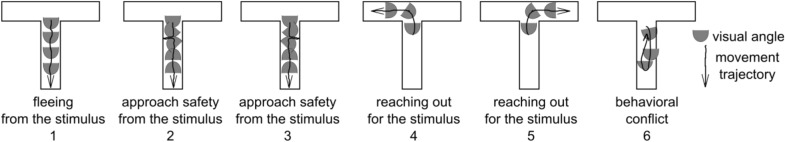
Behavioral categories in the virtual T-maze. The numbers show the different classes that are later used for classification. Note that the behavioral conflict category is not only two different behaviors in one trial but also very bad performance compared to the other trials of the participant. Note also, that the category 2 and 3 are both approach safety behaviors, yet their difference is in the turning direction. This may be interesting dependent on the question on is to answer.

The first problem we wanted to address as an example for the application of a two-layer feedforward network to solve classification problems categorized by experts in the current study is the behavioral classification in a virtual T-maze^2–5^. In the virtual T-maze, humans perform free virtual movement behavior patterns. These patterns used to be categorized into six categories: “fleeing from a stimulus”, “approach safety from a stimulus via left turn”, “approach safety from a stimulus via right turn”, “reaching out for a stimulus via left turn”, “reaching out for a stimulus via right turn”, and “behavioral conflict”. This was performed semi-automatically according to simple rules and was controlled and corrected by hand for every trial^2–5^ (for classification details see Fig. 2 and “Methods” section below). However, as different raters with different experience started to try this classification task, it was evident that additional supervision and training of these raters would be necessary, in order to achieve the same results and accuracy as expert raters. In addition, as more complex behavioral categories arose in some modifications of the paradigm (e.g., conflict, classification 6 in Fig. 2 and “Methods” section below), such a simple classification approach was no longer suitable. Nevertheless, having categorized the behavioral responses of the modified conditions of the virtual T-maze (see Fig. 1 and “Methods” section below) by an expert rater for this task (first author), there was ideal training-, test- and validation-data in order to start the transfer of this problem into an automated classification using two-layer feedforward networks.

The problem that was solved by the two-layer feedforward network was the classification of different behavioral response types in a virtual T maze^3,5^. In this paradigm that is similar to the paradigm used previously^2–5^, the participants experienced five different conditions. Participants were navigating through a virtual T-maze using a joystick. The conditions were linked to credits and cued by color bars at the walls of the T-maze^2–5^. The participants were instructed to go into the maze and earn as many credits as possible^2–5^. They received immediate visual as well as auditory feedback when they gained or lost credits (a pleasant harmonic chord and textual information on credit gain or avoidance of credit loss versus an unpleasant disharmonic chord and textual information on credit loss or miss of credit gain). Also, they experienced a training phase before the experiment, so they were able to react to the different conditions appropriately^2–5^. The duration of one trial was 13 s and 100 Trials (20 per condition) were performed by every participant. In each trial, the participants started in a passage, looking in the direction of the T-arms of the maze and seeing a color cue on the wall of the T-arms of the maze^2–5^. The cue indicated the type of outcomes possible in a trial^2–5^. Participants were able to choose to move further forward towards the T-arms or move away from the T-arms, either by moving backwards or by turning around to move away from the T-arms^2–5^. After the cue had appeared for four seconds, a specific entity (positive trial entity: sheep, negative trial entity: monster, for examples see Fig. 1 or repositories) appeared in the maze^2–5^. The entity was only visible if the participant moved in the appropriate direction in the case of a positive entity running away from the participant, or kept the VR view to the direction were the entity was coming from in case of a negative trial entity^2–5^.

The different types of trials will be described in the following paragraphs and consisted of negative events, positive events, approach-avoidance conflicts, approach-approach conflicts and ambiguous trials^2–5^.

Negative event trials had a monster, chasing the subject and threatening to cause a loss of credits if one could not escape. In response to negative events, one had to escape by not going forward into the T–arms of the maze, but by returning down the starting passage and reaching the starting end of the passage before the monster did. Although one had to go back down to the end of the passage, one could still do so in two ways: moving backwards or turning around and moving forward towards the end of the passage. Negative events were cued with a red color cue on the wall with the position of the cue next to the T–arm of the maze from where the monster approached^2–5^.

Positive event trials had a sheep as entity, providing the opportunity to raise the credits, if one could reach the sheep by moving forward in one of the T-arms of the maze before the sheep reached the end of this passage. Positive events were cued with a green color cue on the wall next to the T–maze arm in which the sheep was running away^2–5^.

The third event category was an approach–avoidance conflict, consisting of a negative and a positive event at the same time, being also cued the same time, but the negative and positive event never being in the same T-maze arm. Having to choose whether they wanted to go forward for the sheep and get caught by the monster, or whether they wanted to flee the monster and miss the sheep, the participants had the same expected outcome value in this trial for both behavioral options^2–5^.

The fourth event was an approach-approach conflict, consisting of two positive events at the same time in different T-maze arms. As the time was not sufficient to get both sheep, the participant had to decide which sheep to follow and which to let go^2–5^.

The fifth event was an ambiguous trial, where the participants saw a yellow cue at one side of the T-maze. This cue indicated that either a sheep or a monster would occur, but the participants were not informed which of those entities they would engage with eventually. Hence, the participants had to decide whether they would go forward in order to possibly catch the sheep, or whether they would run away to escape the monster. Of course, as they did not know whether it would be a sheep or monster, a decision to go after the sheep if the monster was actually present resulted in being caught by the monster and loosing credits, as missing the sheep while running away from a monster that was not present led to a stagnation of the credits.

In order to strengthen the impact of negative and positive outcomes of the events, harmonic and disharmonic chords were presented using headphones together with visual feedback of the credit gain and loss^2–5^. If one reached the sheep that was running away, in addition to the raise of credits, a harmonic chord was played^2–5^. If one did not reach the sheep in time, in addition to the message that the credits remain constant, a disharmonic chord was played^2–5^. If a monster was present and the participant managed to escape the monster, in addition to the message that the credits remain constant a different harmonic chord was played^2–5^. Finally, if a monster was present and the participant was caught by the monster, in addition to the message of the loss of credits a different disharmonic chord was played^2–5^.

At the end of each trial, the participants experienced a white fog, “beaming” them to the starting position for the next trial. A schematic display of the trials and examples for the cueing as well as the entities used in the trials can be seen in Fig. 1.

As the previous behavioral categorization^2–5^ was not fitting anymore, a new categorization was done. All new behavioral categories in these trials explained below were based on the movement and acceleration/deceleration trajectories of the joystick in the virtual T-maze and the time scale, as well as the overall performance of the participant. The manual criteria for the training set that have been selected based on successful reliable empirical classification experience of the expert from the earlier studies are as follows: The first category was fleeing, with the backward movement speed being higher than 75% of the maximum backward movement speed and the turning of the participant being indicated by the joystick excursion to the left or right sides being smaller than 50% of the possible excursion in the first 8 s (Fig. 2). Note that it is important for the first category, that the person is moving backwards and the joystick is not moved further than 50% sideways. As the participants tend to not perform only a straight line while moving backwards, a rather broad margin of error is provided by the 50% yet indicating that there is still no turning around in the relevant time of behavioral classification, while the backward movement is still present. The second and third categories were the approach safety behavior that was characterized by the turning around of the participant indicated by the joystick excursion to the left or right side being higher than 15% and having a change from backward to forward movement in the first 8 s (Fig. 2). Here the specific behavior of changing from backward to forward movement is important and that this is accompanied by a sideway motion of the joystick indicating a turning motion. As the virtual space that the participants may cover during the relevant time window is rather large, a sideway turning of 15% already provides the opportunity to change movement directions from backwards to forwards movement. The reason for two categories was the direction of the turning movement with a distinction between right and left turning. Depending on the specific research question, it might either make sense to combine those two categories eventually into an approach safety behavior category independent of right or left turning or to analyze the two categories separately. It might for example be of interest, whether individual participant characteristics such as handedness influence the preferred turning direction when approach safety behavior is executed or whether the turning movement direction when executing approach safety behavior is related to the occurrence location of the negative stimulus. The fourth and fifth categories were the reaching out movements. They were characterized by the forward movement, indicated by the joystick excursion in forward direction being higher than 50% of the maximum excursion during the first 8 s and the backward movement, indicated by the joystick excursion in the backward direction being smaller than 10% in the first 4 s (Fig. 2). The reason for 2 categories was again the direction of turning. The last category was the most complicated to classify, the behavioral conflict category. This category was picked if the behavioral reaction onset latency was higher than 1.5 s or if there was a change in the behavior during the trial. For instance, if the participants started to flee but then decided to reach out for the stimulus after a short period of time (Fig. 2). Also, this category was chosen if the behavioral performance of the trial was very bad in comparison to other trials executed by the participant, indicating a less accurate execution of the joystick behavior (Fig. 2). These behavioral categories were used to train the two-layer pattern classifier in order to automatize the classification for other experiments that are to come in this virtual T-maze.

### Classification problem example 2: automatic IC artifact detection in EEG research

**Figure 3:**
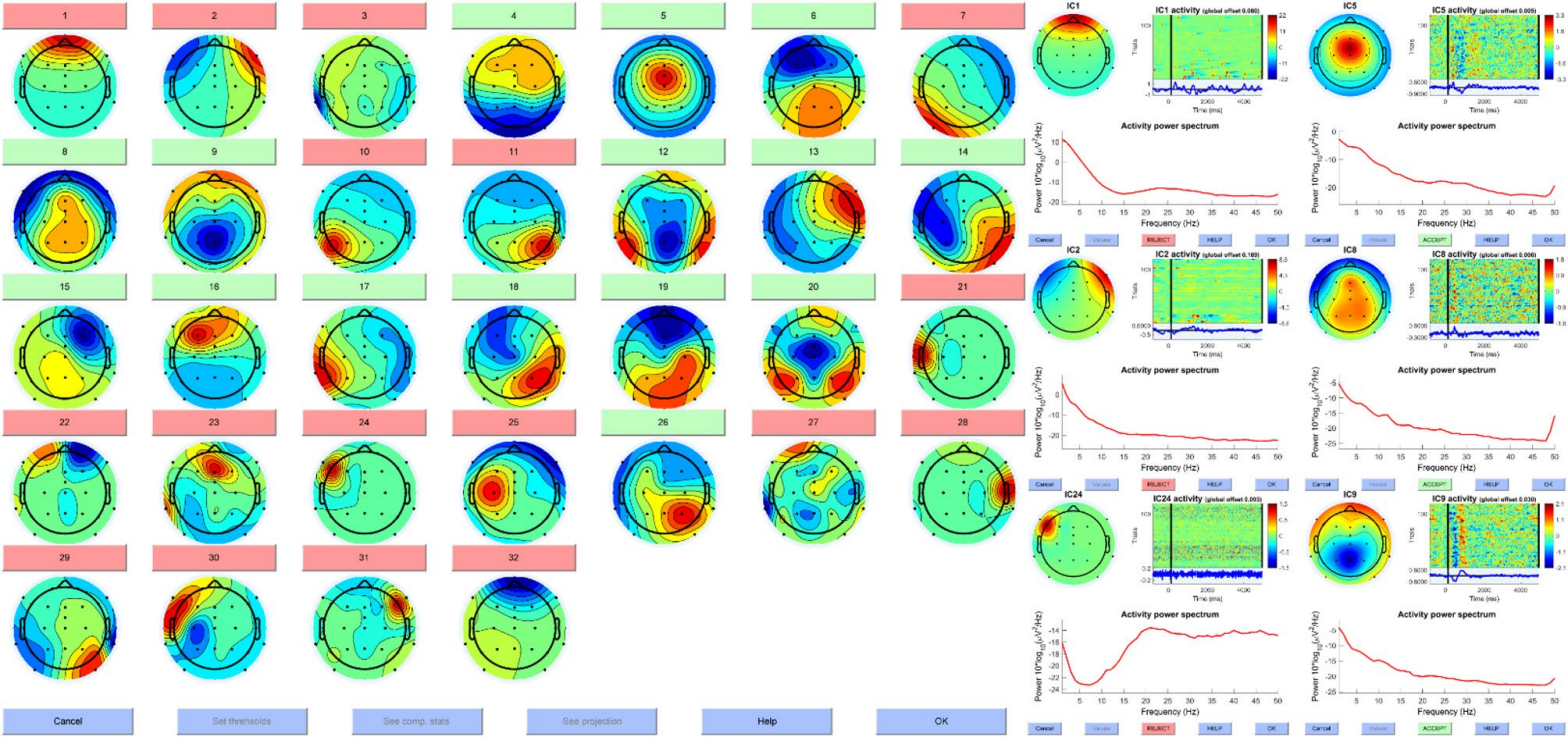
Independent components classified in artifact components (red) and signal components (green). Note in the right panel the frequency distribution, topography and time courses of different artifact and signal components.

The second problem solved with the two-layer feedforward network was the automatic classification of independent component artifact components in the experimental task of the T-maze that produced rather specific artifacts. In EEG research, artifact detection, as exemplarily the detection of muscular signals, pulse related confounds (e.g., from templar structures), eye blinks, eye-movements, electrode site disconnections and other non-brain-related electrical artifacts, is very important to get to a valid and reliable interpretation of the electro-cortical signal. One approach to detect these artifacts is the independent component decomposition (ICA^37^) of the signal, in order to identify underlying signal patterns that are related to artifacts. As the task of detecting an artifact component is a rather complex procedure and not easily explained or performed, researchers needed to be trained for a long time in this process by experts, in order to identify artifact components by the complex combination of topography, time-course and frequency distribution of the component (see example Fig. 3).

Early endeavors to achieve a standardization was correlating the ocular activity with independent components (ICs) and excluded the component that correlated the most with the ocular activation^38^. However as the ocular activation is able to show up in many components due to the very significant structure of this muscular activation and dipole induction, this approach was soon followed by other ideas of standardized artifact IC detection, based on automated algorithms including the temporal and special distribution of ICs^39^ but with the caveat of still having to supervise the detection by human experts^40^. One approach was also to use machine learning algorithms to identify an automatic solution for a specific preprocessing of the data, including the frequency spectrum as an additional information, but excluding high frequency noise above 50 Hz^17^. As these higher frequencies however are rather important to detect several types of artifacts such as pulse artifacts and muscular activity, the provided solution is not ideal for every task, especially if more muscular artifacts are to be expected from behavior that is not only executed by simple button presses, but by other behavioral options including virtual or real movements.

A common theme in all these approaches to classify artifacts via ICs is the idea, that one solution may be used for all tasks at hand. For experimental tasks that are rather similar concerning the appearing artifacts, a good performance of these solutions could also be shown^17,39,40^. On the other hand, if a task produces other types of artifacts, for example due to the response opportunities or the sensual input for the participant, different solutions that are more specific for this type of task will be needed. As the first author of this manuscript has been extensively trained in detecting artifact ICs and applied this knowledge to his research by manually detecting such components in the first place, we used the EEG-activation in the virtual T-maze task explained above as an additional example for the creation of a specific automatic detection mechanism based on expert knowledge. Although different types of artifacts can be detected (see Fig. 3), the prime goal was to detect whether an artifact is present or not. This is the reason why the neural net only has two resulting categories: Signal and Artifact.

### Structure of the neural net: two-layer feedforward network

**Figure 4:**
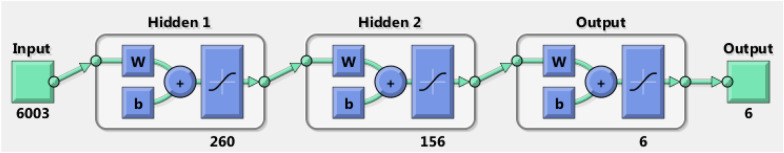
Structure of the two-layer pattern classifier of the first example.

**Figure 5:**
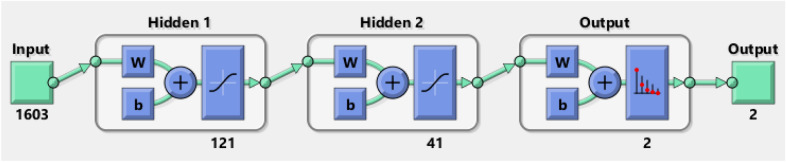
Structure of the two-layer pattern classifier of the second example.

The structure of the nets was determined according to the problem at hand and due to the formula given by Huang^1^ and can be seen in Figs. 4 and 5. The formula given for layer one is $\sqrt{\left(m+2\right)N}+2\sqrt{N/(m+2)}$. The formula for layer two is $m\sqrt{N/(m+2)}$. Here, *N* is the number of distinct input samples and m is the number of output neurons.

### Classification problem example 1: behavior in virtual T-maze

The input vector structure of the neuronal net for the first example consisted of 6003 elements, with 3000 elements being the coordinates in the x direction, 3000 elements being the coordinates in the y direction, the id number of the participant, the trial of the condition and condition number. For the output vector structure, we used six binary elements, classifying all behavioral options specified above. The neural networks were trained with 54 participants with 100 trials each (20 per condition), leading to 5400 different input vectors and a 6003 × 5400 input matrix for the training and determination of the performance. The input vectors and the output vector structure were used to compute the necessary two-layer feedforward network to solve the classification problem. According to the formula given by Huang^1^, the first layer consisted of $\sqrt{(}(7+2)*5400)+ 2* \sqrt{\frac{5400}{7+2}} = 259.81\approx 260$ neurons. The second layer consisted of $7* \sqrt{\frac{5400}{7+2}}=155.88 \approx 156$ neurons. The differentiation of the training set, the validation set and the test set was always kept at 70% training data, 15% validation data and 15% test data. The necessary validation trials for the different neural networks were set from 100 to 150 to 200 trials. This means that either 100, 150 or 200 trials had to be classified correctly in a row to validate the neural network being finished with its internal adjustments of weights. As more validation trials may lead to overfitting of the neural network, the restriction to a maximum of 200 consecutive correct classified trials was used. Two different training algorithms were used in order to compare their performance. The first training algorithm was the “trainscg” that updates the weights and bias values according to the scaled conjugate gradient method^41^, the second algorithm was the “trainrp” that updates the weights and bias values according to the resilient backpropagation algorithm (Rprop^42,43^). The best nets were detected with regard to the overall fit of the classifications in the test and validation part of the set. Additionally, a new dataset of 111 participants plus another new dataset with 62 participants and 100 trials each was categorized by the selected nets and the net with the best performance in this new data set concerning the classification above chance was finally selected (Table 1, chance level for 6 categories: 1/6 = 16.66%).

**Table 1:** Overall performance accuracy, validation trials, algorithms and trials which could not be categorized in the two new datasets for different two-layer feedforward networks.

Two-layer pattern classifiers	Overall performance: classification accuracy	Validation trials	Algorithm	Trials missed in new dataset1 (chance level 0.17)	Trials missed in new dataset2 (chance level 0.17)
net1	98.2	100	trainscg	3	2
net2	97.9	150	trainscg	16	4
net3	98.4	200	trainscg	10	5
net4	96.4	100	trainrp	239	153
net5	97.1	150	trainrp	85	56
net6	96.2	200	trainrp	258	162

The networks were composed of 260 neurons in the first layer and 156 in the second layer.

Net1 has finally been selected. The behavioral categories were selected against the threshold of *p* = 0.17, meaning that if the indicated probability of a behavioral category exceeded this threshold, this category was selected. This specific threshold was selected because of the chance level of selecting a category against 6 possible categories was *p* = 0.1667 and rounded upto *p* = 0.17.

### Classification problem example 2: automatic IC artifact detection in EEG research.

The input vector structure of the neuronal net for the second example consisted of 1603 elements, with 1500 elements being the mean time-course of the component, 32 elements being the topographical information of the component (here for a 32 electrode EEG montage), 70 elements being the frequency band information of the component (from 1 to 70 Hz) and one element being the participant number. For the output vector structure, we used two binary elements, classifying whether an artifact or clean signal is given in the component. The neural networks were trained with 51 participants with 32 components each, leading to 1632 different input vectors and a 1603 × 1632 input matrix for the training and determination of the performance. The input vectors and the output vector structure were used to compute the necessary two-layer feedforward network to solve the classification problem. According to the formula given by Huang^1^, the first layer consisted of $\sqrt{(}(2+2)*1632)+ 2* \sqrt{\frac{1632}{2+2}} = 120.19\approx 121$ neurons. The second layer consisted of $2* \sqrt{\frac{1632}{2+2}}=40.39 \approx 41$ neurons. As in the first example, the differentiation of the training set, the validation set and the test set was always kept at 70% training data, 15% validation data and 15% test data. Also, the necessary validation trials for the different neural networks were set from 100 to 150 to 200 trials and the two different training algorithms “trainscg” and “trainrp” were used in order to compare their performance. The best nets were detected with regard to the overall fit of the classifications in the test and validation part of the set. Additionally, a new dataset of 52 participants and 32 components of resting EEG was categorized by the selected nets.

### Software

The neural networks were made with matlab 2011b and the neural network toolbox Version 7.0.2 for the first example and with matlab R2015b and the neural network toolbox Version 8.4 for the second example. The virtual T-maze paradigm was designed with a freely available game engine^44^, and the participants’ movement and joystick input were acquired using the VR-experimentation software CyberSession CS-Research 5.6^45^. EEG was recorded from 32 electrode positions using BrainVision BrainAmp Standard and Brain Vision Recorder software. For further computation, MATLAB and EEGLAB toolbox^46^ were used.

## Results

### Classification problem example 1: behavior in virtual T-maze

The different two-layer feedforward networks were compared using their overall classification performance. All pattern classifiers performed very well and achieved classification accuracy from 96.2 to 98.4% (Table 1).

**Figure 6:**
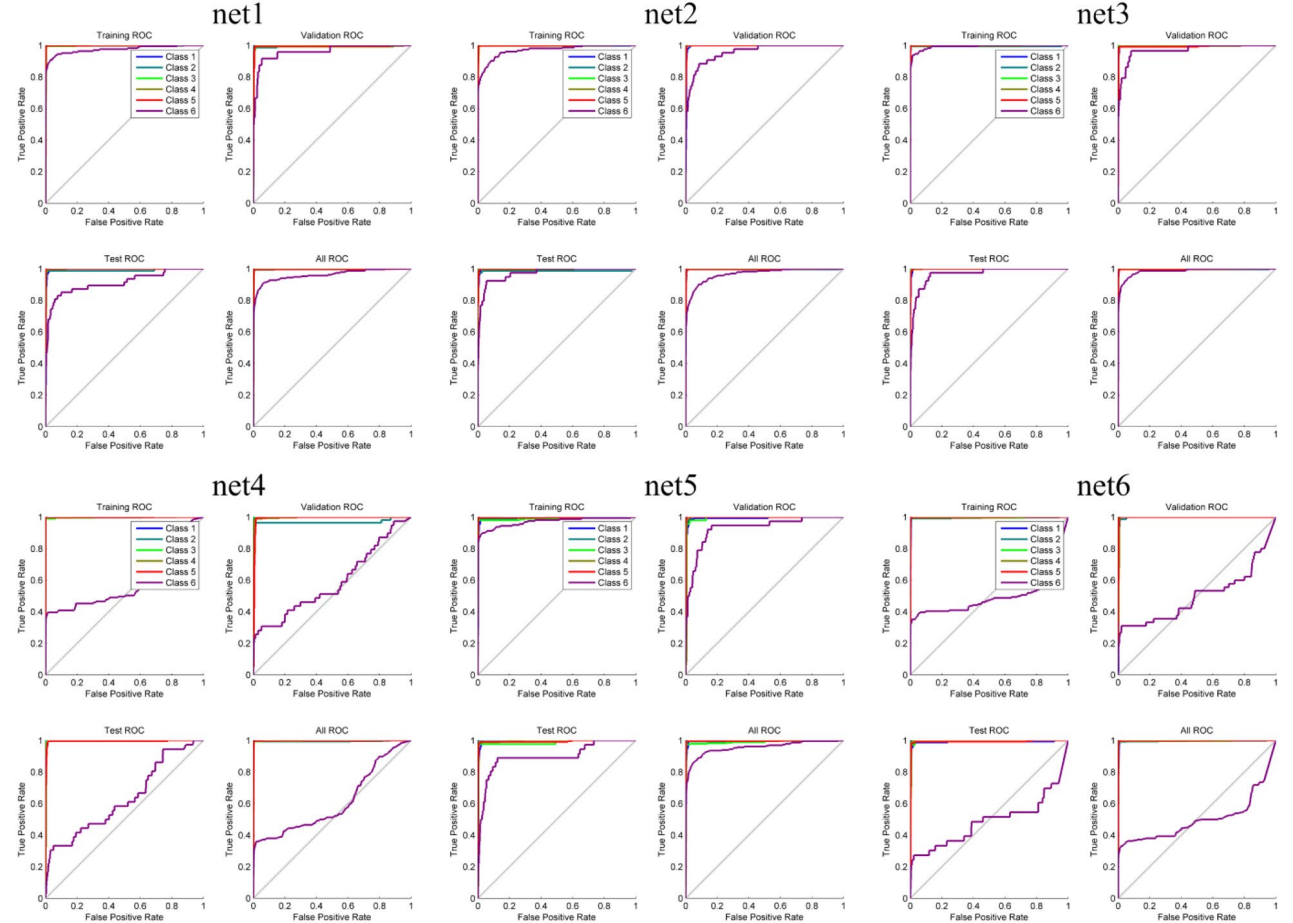
Receiver operating characteristic for the different neural networks. The class number of the different behavioral categories can be seen in Fig. 2.

However, there were substantial differences in the classification of the conflict category (class 6, Fig. 6) and a subsequent classification of a new dataset from the same virtual T-maze (Table 1). The ROC graphs (e.g. Fawcett, 2006) that can be seen in Fig. 6 have the $"\text{true positive rate}" \approx \frac{\text{positives correctly classified as positives }}{total\, true\, positives}$ on the y axis and the $"\text{false positive rate}" \approx \frac{\text{negatives incorrectly classified as positives} }{total \,true \,negatives}$ on the x axis. Hence the ROC graph depicts the relative tradeoffs between benefits (true positive) and costs (false positive). As a perfect classification is reached if only true positives are classified, curves that tend towards the upper left corner of the ROC graphs depict more accurate classifications and therefore better classification performance, as can be seen for net1, net2, net3 and net 5. If a curve is pointing towards the lower right corner of the graph, it indicates that the misclassification is prevalent, as it can be seen in net 4 and especially in net 6.

Based on their overall classification performance Χ^2^_McNemar performance of two best networks compared to others_ > 9.529, *p* < 0.01 and the very low count of missed trials in the new dataset, the two neural networks net1 and net3 were selected for a more detailed comparison.

**Figure 7:**
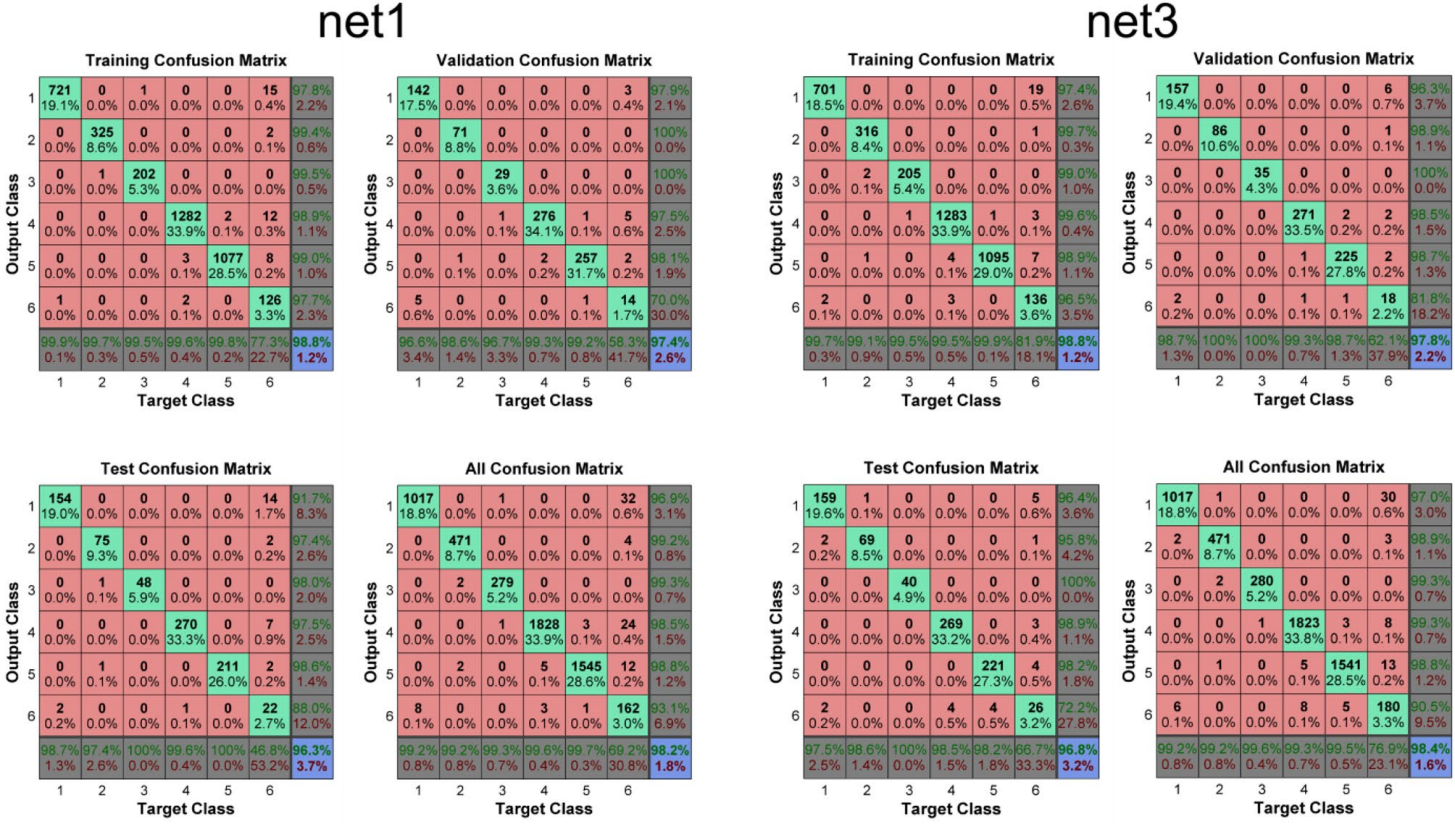
Confusion matrices of the different two-layer feedforward networks. The class number of the different behavioral categories can be seen in Fig. 2.

The confusion matrices and additional information for net1 and net3 are displayed below in Fig. 7 and Table 2. The confusion matrices^47^ depict the $"\text{precision"}\approx \frac{\text{positives correcty classified as positives}}{\text{total classification as positives}}$ in the right column and the true positive rate in the bottom row for the training set, the validation set, the test set and overall for the two different two-layer feedforward networks. Table 2 depicts the true positives, false positives, false negatives and true negatives of every behavioral category along with the false positive rate, the true positive rate, precision and $"\text{accuracy"}\approx \frac{\text{positives correctly classified and negatives correctly classified}}{\text{all cases}}$^48^.

**Table 2:** Common performance metrics calculated from confusion matrix.

Neural network	Behavioral category	True positive	False positive	False negative	True negative	False positive rate (%)	True positive rate (%)	Precision (%)	Accuracy (%)
net1	1	1017	33	8	4338	0.8	99.2	96.9	99.2
	2	471	4	4	4917	0.1	99.2	99.2	99.9
	3	279	2	2	5113	0.0	99.3	99.3	99.9
	4	1828	28	8	3532	0.8	99.6	98.5	99.3
	5	1545	19	4	3828	0.5	99.7	98.8	99.6
	6	162	12	72	5150	0.2	69.2	93.1	98.4
net3	1	1017	31	8	4340	0.7	99.2	97.0	99.3
	2	471	5	3	4917	0.1	99.4	98.9	99.9
	3	280	2	1	5113	0.0	99.6	99.3	99.9
	4	1823	12	13	3548	0.3	99.3	99.3	99.5
	5	1541	19	8	3828	0.5	99.5	98.8	99.5
	6	180	19	54	5143	0.4	76.9	90.5	98.6

Taken together all the information and comparisons of the two neural networks^49^, we decided to use the net1 for further classification of the behavior in the paradigm described above, although no clear decision was indicated by the statistical tests: As the overall classification accuracies of the two neural networks were only marginally different (net1: 98.2%, net3: 98.4%, Χ^2^_McNemar performance net1 vs. net3_ = 2.778, *p* = 0.096), we also had a look on the missing in classification of an entirely new and similar dataset from the paradigm (see Table 1). Still, this comparison was not conclusive for a decision between the two solutions (net1: 3 missed trials, net3: 10 missed trials, Χ^2^_McNemar net1 vs. net3_ = 3.769, *p* = 0.052). Finally, we decided to use net1 because of the marginally significant effect of less missed trials in the classification of the new dataset compared to the marginal effect of overall performance, along with the less validation trials boundary that was set for net1 (Table 1). As the final selection of the neural network of interest was more based on the very low count of missed trials in the new dataset than the overall performance, this decision may of course be debated and net3 may also be selected as best fitting neural network, although it may also be slightly more prone to overfitting because of more validation trials.

### Classification problem example 2: automatic IC artifact detection in EEG research

The different two-layer feedforward networks were compared using their overall classification performance. All pattern classifiers performed rather well and achieved classification accuracy from 88.8 to 94.6% (Table 3). The resting EEG was successfully categorized by all selected nets.

**Table 3:** Overall performance accuracy, validation trials, algorithms and trials which could not be categorized in the resting dataset for different two-layer feedforward networks.

Two-layer pattern classifiers	Overall performance: classification accuracy	Validation trials	Algorithm	Trials missed in resting data set (chance level 0.50)
net1B	94.6	100	trainscg	0
net2B	92.1	150	trainscg	0
net3B	91.8	200	trainscg	0
net4B	92.0	100	trainrp	0
net5B	91.4	150	trainrp	0
net6B	88.8	200	trainrp	0

The networks were composed of 121 neurons in the first layer and 41 in the second layer.

Net1B has finally been selected.

**Figure 8:**
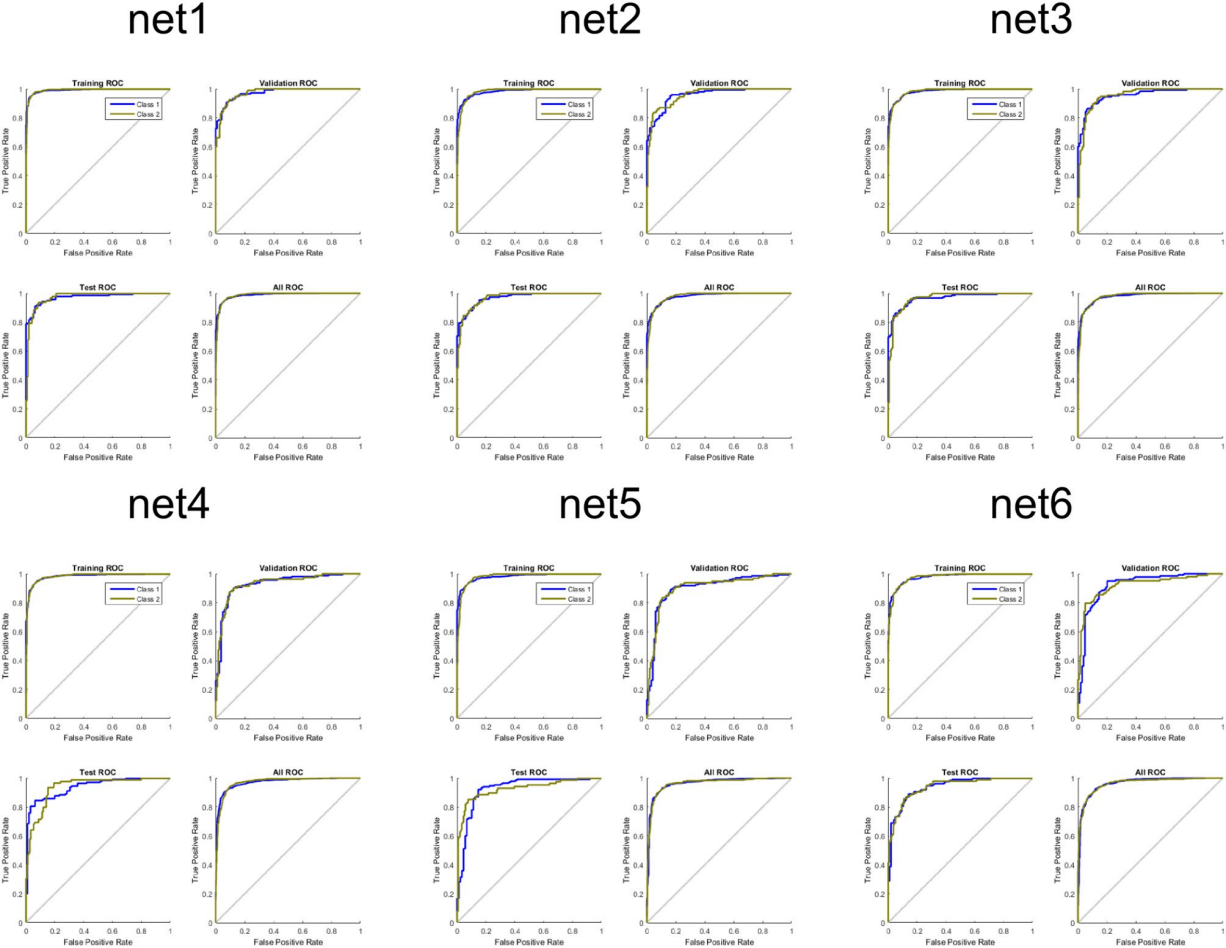
Receiver operating characteristic for the different neural networks. Class 1 means artifacts, Class 2 means signal.

The ROC graphs can be seen in Fig. 8. All nets perform rather well, although the backpropagation algorithm networks perform slightly worse than the other networks.

Based on the overall classification performance Χ^2^_McNemar performance of net1B to others_ > 41.00, *ps* < 0.001, net1B was chosen, although the easy classification task of the resting EEG was solved by all neural networks. Details of all neural networks are depicted in Table 4.

**Table 4:** Common performance metrics calculated from confusion matrix.

Neural network	Component type	True positive	False positive	False negative	True negative	False positive rate (%)	True positive rate (%)	Precision (%)	Accuracy (%)
net1B	Artifact	967	49	39	577	7.8	96.1	95.2	94.6
	Signal	577	39	49	967	3.9	92.2	93.7	94.6
net2B	Artifact	945	68	61	558	10.9	93.9	93.3	92.1
	Signal	558	61	68	945	6.1	89.1	90.1	92.1
net3B	Artifact	937	65	69	561	10.4	93.1	93.5	91.8
	Signal	561	69	65	937	6.9	89.6	89.0	91.8
net4B	Artifact	938	62	68	564	9.9	93.2	93.8	92.0
	Signal	564	68	62	938	6.8	90.1	89.2	92.0
net5B	Artifact	929	64	77	562	10.2	92.3	93.6	91.4
	Signal	562	77	64	929	7.7	89.8	87.9	91.4
net6B	Artifact	876	53	130	573	8.5	87.1	94.3	88.8
	Signal	573	130	53	876	12.9	91.5	81.5	88.8

To sum it up, in this example the net1B performed very well with the best accuracy, although all other networks created here were also able to classify the task of resting EEG without any omissions.

## Discussion

In this work, we showed that it is possible to create neural networks to classify continuous behavior in a task as well as perform ICA artifact classification as examples for manual tasks that were previously categorized by experts. We used two-layer feedforward networks to classify our examples for any expert categorization decision that can be solved with a reasonable input vector structure. The structure of the net was computed using the formula given by Huang^1^ and this formula led to effective results concerning the fitting of the different neural networks. Nevertheless, the results show that it is important to test the different neural networks for their performance on new data in order to see whether this data can be categorized in meaningful ways and no overfitting is given. As we tested two different training algorithms and therefore propagation rules, we found that the resilient backpropagation algorithm (net4, net 5, net 6) led to less favorable results than the scaled conjugate gradient method (net1, net2, net3), as one would expect from benchmark tests^50^ as well as that the architecture given by Huang^1^ was intended for feedforward propagation. This leads to the assumption that the resilient backpropagation as it is implemented in matlab 2011b and the nntoolbox Version 7.0.2 might not be the optimal algorithm to be used in the two-layer feedforward networks, or at least in this specific problem. However, this result has also been replicated by our workgroup^5^, so we believe this finding to be fairly robust. Also, the benchmark tests suggest that the scaled conjugate gradient algorithm performs well over a variety of classification problems^50^. Another interesting result was that the network with the best ROC curve and confusion matrix concerning the input vectors (net3) did not perform best on the new data, but a network that was very close to this performance (net1). This may indicate that the best performing net can already be prone to overfitting, leading to a too specific solution of the classification and therefore to problems with the new data. This makes the testing with new data a very important step of the validation process of the automatic classification. However, the difference in performance on the trained and new dataset between the two neural networks is rather small and one might argue whether the preference of net1 vs. net3 is a necessary choice. Nevertheless, we chose net1 as we were interested in the classification of as many behavioral trials as possible with a reasonable solution, also on new data that is still to come. As the importance of losing trials or having a potential overfitting might be very different in other cases, other researchers might prefer solutions that are similar to net3 in their specific case. Nevertheless, one conclusion can be drawn from these results: it is still important to perform tests of the neural networks on new data and it is important to check whether the network that was chosen is able to categorize the data sufficiently accurate.

For our second example, there was no difference in the performance of the created neural networks, in a slightly easier task than the nets were created for. This indicates that the difficulty of the problem used to create the net should match the difficulty of the new data sets if possible, if no other criteria than the classification omission can be used (fortunately, there is a significant performance difference that we could use to select net1B as explained above). Having created neural networks for behavioral responses as well as for IC artifact detection in this virtual T-maze task, we have achieved solutions for two problems that used to be solved by expert decisions. Now, these solutions can be used by other researchers in order to get a standardized method that can be applied by anyone to these problems. Thus, we can get more reliable and replicable results in this kind of behavioral classification as well as in this specific IC artifact detection task.

### Seven questions about the two-layer feedforward networks and their impact on the field

Having reiterated the findings for the examples in detail, we may ask important questions:

1. **How to create a neural network for the given problem?**
  Huang^1^ provides a detailed instruction to create neural network for classification than can be used for every task where a meaningful input vector can be created and where an expert has already taken an approach on the classification. This does not imply that the entire task is already categorized, but a sufficient learning set for the task should be given. The architecture of the net is dependent on the sample size and output vector, as mentioned in the “Methods” section above.
2. **Is the neural network “better” than the expert that was used to create it?**
  This question cannot be answered easily, but there are different perspectives on that question. First, one may say that the neural net can be shared with other researchers and therefore this expert opinion can be shared without having to interpret or even trying to teach this kind of expertise. This enables many researchers to use the expertise without the expert having to perform every time. Also, the time used to classify the behavior by hand would be several days or weeks, while the neural net performs it in seconds or minutes. Hence, we get a faster and widely shared version of the expert´s opinion. Nevertheless, every bias that was consistently present in the expert is now transferred to the neural network. The net merely reproduces the classification without variable biases that may have been introduced by different states, but the consistent biases of the expert are still present. This is very important to keep in mind, as the network does not guarantee a correct classification (as is often seen by neural networks being “racists” in case of threat detection or income- and work-related questions). It is only a generalization of the learned classification that can be used to reliably and consistently classify the data. Also, the experts are able to adapt their judgements to new types of classes that may arise in new datasets (for example a new type of behavior showing up or a new class of IC artifact due to a different EEG-setup), while the net will not be able to do so. Hence new types of classes will not be detected and classified in a wrong manner (see e.g., pulse related artifacts from templar structures in MARA for EEG IC artifact classification).
3. **Is one neural network enough to classify a task?**
  While the architecture provided by Huang^1^ is very promising to create neuronal networks to replicate every classification solution by experts, it is still important to check what differences are given by the differences of the learning rule and the propagation restrictions, as well as the training and validation sampling size. Still, not only the validation in the sense of the machine learning sample error needs to be evaluated, but also the applicability to new datasets in order to identify well-fitting networks that are not over-fitted to the respective training data. Hence, it is important to create different networks with slightly different training samples in order to select the neural network that provides a fitting combination of the least overfitting and the best classification performance.
4. **Are different neural networks needed for every single task?**
  If the tasks are similar, with identical output and input categories, then a new neuronal net will not be needed. Otherwise, one needs to create a different neural network or at least check the results concerning plausibility as well as for classification accuracy and uncategorized cases. If the input vector size is different, a new net will be needed.
5. **How to create the input vector and output vector?**
  This question is essential in order to create a successful neural network and is not easy to be answered. The output vector should be your classification results as logical/binary vectors. The count of the output nods is the count of your classes. However, for the input vector, the structure is not that easy to determine. You have to identify necessary information that the expert uses to create the classification and exclude information that is irrelevant in order to get a good performing neural network for the problem. In our first example, this meant not including all data from the entire behavioral performance, but only the relevant time point that we also used for manual classification. For the second example, this was even more restrictive, as an input vector of 120 (trials) * 1500 (datapoints EEG segment) = 180,000 datapoints would have been possible, but most of the data would not have given any information about the classification. As the manual task performed by the expert is also based on the frequency distribution, the topography and the mean time course of the component, we identified these features as a meaningful input vector in this case.
  This task to identify the input and output vector is very important and it takes an expert to identify the needed features of your task for a successful automatic classification.
6. **Will experts become “worthless” as their opinion can be shared efficiently?**
  No! As explained above, experts provide meaningful classification sets, the evaluation of features that can be used to create the architecture of the neural network as well as the assessment whether the neuronal networks are able to perform adequately and without any performance errors in the classification of data. Their expertise will be the foundations for other researchers to build their neural networks on and therefore their work is very important and should be valued.
7. **How may this standardization of data classification have impact on our field?**

Hopefully, the standardization and availability of these neural networks to complex decisions can lead to a more replicable and reliable research in our field. Complex tasks such as behavioral categorization in animal research, automatic picture classification, movement decision categorization according to continuous data or complex physiological patterns that were only categorized by experts may now be solved by different experts using their trained neural networks. As every expert may train a different net or even more experts may train a net on their consent, different replicable classifications for complex data may arise and can be compared. This does not only provide the basis of reliable and comprehensible research, but also may provide insights into the differences in expert opinions as one may choose to apply different neural networks to the data and compare those resulting classifications. A productive discussion can arise from these comparisons, as perspectives of these different experts may not only be debated on the theoretical level, but also on different concrete datasets. Therefore, not only a general reliability and more replicable science may rise, but also the experts may further discuss the specificity of their classifications and the appropriate expert solution may be applied to the data at hand.

As mentioned above, a similar approach has already been taken for other problems too^11,12^ and in theory, one might be able to construct a two-layer feedforward neural network for virtually every classification problem^1^. Yet it has to be noted that deep learning approaches including recurrent structures^23^ or multilayer networks, are better suited for certain more complex problems, especially for modeling visual pattern recognition^21^. Neural networks that can be provided to other researchers do not only lead to a quick solution for problems at hand, but they also provide a standardized solution instead of introducing correction by eye or other methods that are hard to replicate. Huang^1^ provides a very easy formula to construct two-layer feedforward neural networks to address virtually every categorization problem and with the present work we try to encourage other researchers to also work on neural networks solving tasks that are yet done by hand, in order to standardize the results. Although experts might get sometimes the impression of “losing their expertise to others” by publishing and providing their expertise to other via these neural networks, it is important to convince them that they can help the field to advance if they contribute their knowledge in training and creating neural networks for the whole scientific community.

In summary, in order to get to a good and reliable science, it is important to not only publish interesting and marvelous results, but also to provide the scientific community with the necessary tools to solve problems in standardized ways^51^. Using and applying the work of Huang^1^ we may build standardized classification tools for many problems as shown here for the examples of the behavioral classification of continuous movements in the virtual T-maze and the IC-artifact detection in the same task. Hence, we may be able to gradually work our way to a reliable and sharing scientific community, tackling replication problems and opening a new level of expert discussions as more datasets may be classified by different expert approaches.

## Data Availability

The data of the examples as well as the paradigm (VR-T-Maze) are available from the following repositories. Repositories: FTP: http://update.cybersession.info/T-Maze/VrProject.T-Maze.zip (282 MB, 2019-10-15); http://update.cybersession.info/T-Maze/TwoLayerFeedForward_Data.zip (330 MB). Repositories: SVN: https://crm.vtplus.eu/svn/open-access.t-maze/trunk; https://crm.vtplus.eu/svn/open-access.t-maze/trunk/Paradigm; https://crm.vtplus.eu/svn/open-access.t-maze/trunk/sourcemods/VrSessionMod06_T-maze.
